# Assessing the role of sustainability competencies in enhancing psychological first aid effectiveness for disaster responders in Fiji

**DOI:** 10.3389/fpubh.2024.1349342

**Published:** 2024-06-26

**Authors:** Malini Nair, Serik Meirmanov

**Affiliations:** ^1^Graduate School of Asia Pacific Studies Doctoral Degree Program, Ritsumeikan Asia Pacific University, Beppu, Japan; ^2^College of Sustainability and Tourism, Ritsumeikan Asia Pacific University, Beppu, Japan

**Keywords:** psychological first aid, sustainability, competencies, learning outcomes, effectiveness, disaster responders, Fiji

## Abstract

**Background:**

Psychological first aid (PFA) is essential for mental health and wellbeing after traumatic events. Integrating competency-based outcomes is crucial with the increasing demand for effective psychological first-aid interventions. This study examines the correlation between sustainability competencies and PFA principles within Fiji's disaster responder's context.

**Method:**

The research was guided by a theoretical framework based on a comprehensive review of sustainability competencies and PFA principles. A cross-sectional survey assessed the importance of sustainability competencies in disaster responders to deliver PFA effectively. The survey used a stratified random sampling method to get diverse PFA-trained participants (66%) and non-PFA trained (34%), aiming to understand how these competencies can impact PFA success in various disaster situations. The survey, encompassing various domains of disaster response and a diverse range of respondents age, gender, and years of experience, employed the Likert scale to assess the importance of competencies such as integrated problem-solving, strategic, systems thinking, self-awareness, normative, collaboration, anticipatory, and critical thinking.

**Results:**

The study involved 49 PFA-trained participants (55% female, 45% male) and 15 non-PFA-trained participants (53% female, 46% male), excluding 10 responses from the latter group due to ambiguous answers to critical questions. The correlation between age, experience, and the valuation of professional competencies among disaster responders indicates that disaster responders, with extensive experience and PFA training, rated competencies as “important,” reflecting a perspective shaped by long-term career development and practical experiences. Equally, younger and early career responders emphasize competencies as “very important,” indicating an initial recognition of their significance. The appraisal patterns across different age groups, especially among those with PFA training, suggest a tendency to moderate assessments of competency importance with increasing experience. Statistical analysis, including mean, median, standard deviation, and variance, provided a detailed understanding of the data, underscoring competencies like self-awareness in both data sets and integrated problem-solving and collaboration within PFA-trained responders as the key for effective PFA interventions.

**Conclusion:**

The study underlines the critical need to integrate sustainability competencies into the PFA curriculum in Fiji's unique sociocultural context. This interplay between age, experience, and competency assessment stresses the diverse factors influencing perceptions in the disaster response field beyond experience alone. The results show that sustainability competencies are the ultimate to the effectiveness of PFA measurement and interventions. The research lays the foundation for future studies to develop validated tools for assessing sustainable competencies in different cultural contexts, thereby improving the effectiveness of PFA in disaster management. Integrating these competencies into PFA training could significantly strengthen PFA intervention and competency-based evaluation.

## Introduction

Psychological first aid (PFA) is an essential intervention and a critical mechanism for promoting mental health, wellbeing, and resilience in the aftermath of traumatic events. PFA is seen as a practical, early psychosocial intervention to reduce distress following disasters (1, 2). The increased demand for effective PFA interventions necessitates measurable learning outcomes to guarantee their effectiveness. Early assistance for individuals affected by traumatic events has become vital, especially considering the rise in global disasters and crises. The World Disasters Report 2020 highlighted increased climate- and weather-related disasters, from 76% in the 1960's to 83% in 2010–2019 (3). The data indicates the physical damage caused by these events; however, discussions on psychological consequences are limited (4). Anticipated modifications in climate patterns are expected to spur a change in human settlement trends, a transition that could potentially precipitate significant public health implications. These may include heightened susceptibility to infectious diseases, escalated mental health concerns, restricted access to healthcare services, and the emergent need for comprehensive policies and interventions tailored to address the health requisites of displaced populations (5). The intense climatic disasters in Fiji could notably affect the mental and psychological health of the impacted individuals. Fiji is highly susceptible to climate extremes, such as droughts and heavy precipitation, putting its food, infrastructure, and population at risk. The frequency and intensity of these events have increased over time (6). Being subjected to multiple disasters can put immense stress and emotional strain on individuals and communities, disrupting regular routines, causing the loss of homes or loved ones, and creating uncertainty and unpredictability (3). The displacement, loss of residences, and infrastructure damage might engender instability and trigger severe psychological and emotional effects such as anxiety and distress among the poor urban populace (7). The potential for illness and the interruption of regular activities due to flooding and climate hazards might add to psychological stress and trauma (8). Nonetheless, these articles do not explicitly delve into the mental health effects or resilience strategies tied to the difficulties encountered by Fiji's impoverished communities.

PFA's role is crucial in the context of global disasters, and given this backdrop, the demand for comprehensive PFA programs is evident. PFA training programs have proven beneficial for healthcare personnel by improving their knowledge, confidence, and competence (9). There is a lack of evidence on the effectiveness of PFA and research and evaluation methods are still in development (9), noting that PFA is still evidence-informed (10). There is a lack of research assessing the efficacy of PFA and similar initial intervention models within first responder organizations, primarily due to the difficulties encountered in evaluating early interventions in such settings (11).

PFA's core principles include promoting a sense of self and community efficacy, safety, connectedness, calming, and instilling hope (4, 12). The critical aspects of PFA focus on assessing needs, providing practical care and support, listening without pressing discussions, connecting individuals to information, and offering further support (10). Psychosocial workers require key competencies while helping victims, such as active listening skills, critical observation of the surrounding environment, empathy, effective paraphrasing, and correct recognition of needs (13). Despite PFA's aim to reduce initial distress and enhance short- and long-term adaptive functioning, insufficient conclusive evidence supports its effectiveness (4). Several experts have emphasized the importance of integrating PFA into broader care systems and the need to enhance the skills and abilities of PFA providers by developing their competencies (14, 15) and enhancing competency-based evaluation to ensure continuity and effectiveness (16).

For the professional development of responders, defining and validating competencies is crucial. Competencies such as knowledge, skills, attitudes, and values are essential for first responders' professional development and for appraising interventions' effectiveness (17). Current dimensions of perceived competence in PFA include skills, knowledge, attitudes, and the ability to prevent stress and burnout (18). However, broader competencies such as systems thinking, strategic, anticipatory, critical thinking, normative, collaboration, self-awareness, and integrated problem-solving (19), known as sustainability competencies, could enhance the understanding, capability, and capacity to respond and effectively assess PFA interventions (17, 19). These competencies, which refer to the capacity for delivering persistent and practical reactions to emotional and psychological distress, are essential to the effectiveness of PFA interventions (17, 19).

It is imperative to evaluate the effectiveness of PFA and its associated competencies within a robust monitoring and evaluation framework as part of disaster response efforts. This assessment is crucial to ascertain whether the training aligns with and fulfills the specific requirements and characteristics for disaster responders' performance (11). Evaluating the efficacy of PFA requires understanding the sustainable competencies underpinning its implementation. However, there is no consensus on a uniform definition of sustainable competencies in mental health, particularly concerning PFA. The lack of a standardized evaluation framework challenges and complicates the measurement and assessment of the effectiveness of PFA (15). Previous studies have not fully addressed all eight sustainable competencies and the multidimensional nature of these competencies. Self-report measures, often used in assessing competencies such as knowledge, skills, attributes, and confidence, introduce subjectivity and potential bias (14, 15). Understanding the relationship between PFA and sustainable competencies could contribute to developing more effective learning outcomes and a deeper understanding of how PFA interventions impact mental health outcomes (4, 19).

This study aims to strengthen the assessment and comprehension of PFA interventions by emphasizing the role of enduring competencies. Although substantial research exists on the effectiveness of PFA interventions (1, 2, 4, 9, 15), insufficient emphasis has been placed on the incorporation of lasting competencies as quantifiable outcomes in PFA initiatives. Vital for addressing the intricate challenges of disaster health management and promoting long-term resilience, sustainable competencies, this study aims to encompass systems thinking, anticipatory skills, normative abilities, strategic thinking, collaboration, critical thinking, self-awareness, and integrated problem-solving (19). This research investigates the relationship between PFA principles and sustainable competencies, scrutinizing their correlation with interaction and the potential for these competencies to underpin competency-based outcomes for PFA training and evaluation. By collating disaster responders' perspectives, the study underscores the importance of enduring competencies in PFA learning and assessment, identifies shortcomings, and seeks to boost the overall effectiveness of PFA interventions.

## Theoretical framework for psychological first aid and the correlation of sustainable competencies

This study employed a comprehensive review to delineate the original definitions of PFA principles and sustainability competencies, aiming to comprehend the relationship between them. We based the sustainability competencies on the interpretation of the United Nations Educational, Scientific and Cultural Organization (UNESCO) (17, 19, 20), and aligned with the sustainable development goal (SDG) 4 on quality education. The study references the Inter-Agency Standing Committee (IASC) (12) and the World Health Organization (WHO) (21) for the foundational principles and definitions of the PFA framework.

The correlation between PFA principles and sustainability competencies is not immediately apparent, as one focuses on immediate psychological response and the other on long-term global and environmental outcomes. However, on closer inspection, there are indeed convergences, particularly when considering the broader implications of psychological resilience and adaptive capacity in sustainable development. The theoretical framework for PFA incorporates sustainable competencies essential for effective PFA interventions as demonstrated in Table 1. There is a significant correlation between these competencies, and they provide comprehensive and sustainable support for PFA.

**Table 1:** PFA correlation with sustainability competences and expected learning outcomes.

**Sustainable competencies**	**PFA correlation with sustainable competences**
Systems thinking	Systems thinking involves understanding the complex interplay between factors influencing an individual's mental wellbeing. It enables PFA providers to recognize the interconnectedness of various systems (e.g., social, cultural, and environmental) and their impact on mental health outcomes. PFA interventions can be tailored to address the specific needs of the affected population (15).
Expected learning outcome	The learner will be able to identify and analyze the interconnectedness between different systems (e.g., social, cultural, and environmental) and their impact on mental wellbeing and apply this understanding to identify and address systemic factors contributing to distress.
Anticipatory skills	Anticipatory skills involve identifying potential stressors or triggers that may exacerbate distress. PFA providers with strong anticipatory skills can proactively address these stressors and implement preventive measures. PFA interventions can provide tailored, timely, and practical support by proactively identifying stressors, triggers, and risk factors. PFA providers can potentially reduce the severity and duration of distress experienced by individuals (10, 23).
Expected learning outcomes	The learner will be able to proactively identify potential stressors or triggers in individuals' lives and develop strategies to implement preventive measures to reduce the severity and duration of distress.
Normative	Normative abilities, including an understanding of cultural, ethical, and legal considerations, are essential in evaluating the efficacy of PFA. By respecting diverse norms and values, PFA interventions can ensure cultural appropriateness and promote the acceptance and utilization of psychological support. They can also adapt their interventions to respect and honor diverse perspectives and cultural practices. This competency ensures that PFA interventions are culturally sensitive and empower individuals within their contexts (12).
Expected learning outcomes	The learner will demonstrate knowledge and awareness of cultural, social, and ethical norms that influence mental wellbeing and apply this understanding to adapt interventions to respect and honor diverse perspectives and cultural practices.
Strategic thinking	Strategic thinking involves preparing and implementing effective PFA interventions based on thoroughly evaluating the individual's needs. Providers with strong strategic thinking skills can prioritize interventions, allocate resources efficiently, and adapt their approach as the situation evolves. This competency enhances the overall effectiveness of PFA interventions (24).
Expected learning outcomes	The learner will demonstrate the ability to plan and implement effective PFA interventions based on a thorough assessment of individual needs, prioritize interventions, allocate resources efficiently, and adapt their approach as necessary.
Collaboration	Collaboration involves working effectively with other professionals, stakeholders, and community members to provide holistic and integrated support. PFA providers who engage in collaborative practices can tap into diverse expertise, resources, and perspectives, leading to more comprehensive and sustainable interventions (4).
Expected learning outcomes	The learner will demonstrate practical collaborative skills by working with other professionals, stakeholders, and community members to provide holistic and integrated support, tapping into diverse expertise, resources, and perspectives.
Critical thinking	Critical thinking involves analyzing information, evaluating evidence, and making informed decisions in PFA settings. PFA providers with strong critical thinking skills can assess the effectiveness of interventions, identify gaps in knowledge, and adapt their approach accordingly. This competency promotes evidence-based practice and enhances the quality of PFA interventions (25).
Expected learning outcomes	The learner will demonstrate critical thinking skills by analyzing information, evaluating evidence, and making informed decisions in PFA settings, and applying this thinking to assess the effectiveness of interventions, identify gaps in knowledge, and adapt their approach accordingly.
Self-awareness	Self-awareness refers to the ability to reflect on one's beliefs, biases, and reactions when providing PFA (26). PFA providers with high self-awareness can effectively manage their emotions, maintain professional boundaries, and provide empathetic support. This competency fosters a supportive and non-judgmental environment for individuals seeking PFA (12, 27).
Expected learning outcomes	The learner will demonstrate self-awareness by reflecting on their beliefs, biases, and reactions when providing PFA, effectively managing their emotions, maintaining professional boundaries, and providing empathetic support.
Integrated problem-solving	Integrated problem-solving involves analyzing complex situations, identifying underlying issues, and developing comprehensive solutions. PFA providers with strong integrated problem-solving skills (27) can address individuals' interconnected challenges, considering immediate and long-term needs. This competency ensures that PFA interventions are holistic and sustainable.
Expected learning outcomes	The learner will demonstrate integrated problem-solving skills by analyzing complex situations, identifying underlying issues, developing comprehensive solutions, and applying this approach to address interconnected challenges individuals face, considering immediate and long-term needs.

By incorporating these expected learning outcomes into a PFA curriculum, training programs can equip PFA providers with the necessary competencies to provide practical and comprehensive support to distressed individuals. The learning outcomes mentioned here are general and can be further tailored and expanded based on the specific objectives of the PFA curriculum.

The study underscores a robust correlation between sustainable competencies and the PFA curriculum, thereby accentuating their indispensable role in bolstering the efficacy of PFA practices. Within this framework, Systems Thinking equips PFA providers with the capacity to comprehend the intricate interplay of societal, cultural, and environmental factors (19) impacting mental wellbeing, thus enabling the tailoring of interventions to address these specific influences. Anticipatory skills allow providers to foresee potential stressors and facilitate preventive measures, thereby mitigating the severity and duration of distress. Normative abilities ascertain cultural appropriateness, ethical compliance, and legal consideration in PFA interventions, fostering wider acceptance and utilization of psychological support. Strategic thinking enhances resource allocation optimization, intervention prioritization, and strategy adaptability based on individual needs (19). The collaboration underscores the creation of productive partnerships with other professionals and community members, enabling a more holistic and integrated provision of support. Critical thinking capacitates providers to analyze information, evaluate evidence, discern the effectiveness of interventions, and identify knowledge gaps, thereby endorsing evidence-based practice. Self-awareness advocates for effective management of emotions, maintaining professional boundaries, and providing empathetic support, collectively fostering a nurturing and non-judgmental environment (22). Lastly, Integrated Problem-Solving ensures PFA interventions' holistic and sustainable nature by empowering providers to unravel complex scenarios, identify underlying issues, and craft comprehensive solutions. These competencies augment the quality of PFA interventions and guarantee their sustainability, thereby emphasizing their pivotal role in shaping the PFA curriculum.

## Methodology

### Theoretical framework

This study explored the potential correlation of sustainability competencies and the PFA principles. The initial phase involved constructing a theoretical framework, as detailed in Table 1, through a comprehensive review. This review included various scholarly articles, UNESCO Sustainable Development Goals (SDG) 2030 reports (17, 19, 20), the Inter-Agency Standing Committee (IASC) Manual (12), the World Health Organization's PFA manual (22), and the Johns Hopkins RAPID PFA model (4). We aimed to interpret the synergistic relationship between sustainability competencies and PFA, focusing mainly on identifying and analyzing competency-based learning outcomes and their potential to enhance the effectiveness of PFA intervention. We critically examined each outcome for its relevance and application in PFA practice. Additionally, our study was contextualized within Fiji's unique sociocultural and environmental setting, allowing for the identification of gaps in the current PFA curriculum and the development of context-specific strategies.

### Assessing the importance of sustainability competencies among the Fiji disaster responders

#### Study design and procedure

We conducted a cross-sectional survey targeting disaster responders in Fiji, a South Pacific archipelago comprising over 330 islands, of which ~110 are habitable. Fiji has an estimated population of 890,000 and covers an area of about 18,300 km^2^ (28).

Our survey, conducted from May 12 to July 5, 2023, aimed to assess the importance of sustainability competencies for effectively providing PFA, a psychosocial support activity in disaster response. To ensure a representative sample of respondents, we employed a stratified random sampling approach, systematically distributing online questionnaires to various organizations involved in climatic disaster response across Fiji. The survey encompassed a diverse cohort comprising disaster responders who had undergone PFA training and those without PFA training.

The Likert scale was used to quantify the views of disaster responders regarding the importance of sustainability competencies in their profession. The Likert scale comprised four response options: very important, important, somewhat important, and not at all important. Utilizing a Likert scale facilitated a comprehensive analysis of the responses, allowing for a gradual evaluation of the perceived importance of these competencies.

This approach was formulated to encompass a wide selection of views and experiences by classifying potential respondents based on the following criteria.

**Figure 1:**
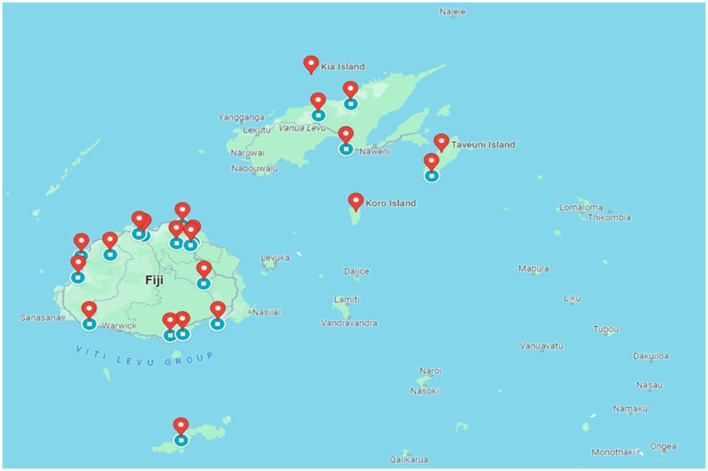
Geographic distribution of disaster responders participating in the survey. (This map was created using Google Maps).

**Area of response and geographic location:** Respondents were categorized according to their specific domain of disaster response operations and their geographical placement within the Fiji archipelago (as depicted in Figure 1).

**Years of experience:** The classification process also considered the breadth of professional experience possessed by disaster responders, which ranged from 0.5 to 33 years, as delineated in Table 2.

**Table 2:** The extremum points for years of experience and midpoint by gender and age.

**Gender**	**Age (years)**	**Extremum points for the years of experience**	**Midpoint**
**Disaster responders with PFA training**			
16–30 years old	1–13 years	7
31–40 years old	3–10 years	6.5
Female	41–50 years old	1–7 years	4
51–60 years old	2–33 years	17.5
16–30 years old	3–5 years	4
31–40 years old	3–8 years	5.5
Male	41–50 years old	2–16 years	9
51–60 years old	7–30 years	18.5
**Disaster responders without PFA training**			
16–30 years old	0–1	0.5
31–40 years old	1–4	2.5
Female	41–50 years old	1–3	2
51–60 years old	0.5–4 years	2.25
16–30 years old	0.5–12 years	6.25
31–40 years old	None	0
Male	41–50 years old	0–5 years	2.5
51–60 years old	0–0.5 years	0.25

**Age group:** Participants were allocated into age brackets from 16 to 60.

**Gender:** Respondents were also distinguished based on their gender (as specified in Table 2).

This approach was fundamental to our methodology to maximize the diversity and representativeness of the survey sample. Such a strategy enhances the reliability and validity of our data, particularly in understanding disaster responder's specific competencies and experiences within the unique environmental and sociocultural context of Fiji. This comprehensive approach helps derive more generalized conclusions about the target population. It provides insights into specific subgroups, enabling a more complex understanding of Fiji disaster responder's roles and challenges.

#### Data collection process

The survey used two platforms to gather the responses. The first platform was the Kobo toolbox for humanitarian surveys, which was developed for disaster responders living in remote communities without internet access and needed basic translation from English to Fijian to understand the study before responding. The link was shared, and entries were done in English. The second platform was Microsoft Forms, which was accessible online through the link shared. Through the surveys, we aimed to gather respondent's answers about sustainable competencies relevant to their work and to indicate the potential impact of these competencies in enhancing their disaster response and recovery efforts.

#### Data analysis

Data were acquired from 74 disaster responders. Researchers further segregated the data into two categories: participants trained in PFA and other psychosocial support activities and those lacking such training. The PFA-trained group comprised 49 participants, with a gender distribution of 55% female and 45% male. In contrast, the group without PFA training comprised 15 participants, featuring a gender distribution of 53% female and 46% male. Additionally, the researchers excluded ten responses from the non-PFA-trained participants due to ambiguous responses to the leading questions. The data was analyzed using Microsoft Excel and further verified using Python version 3.11.4 to adjust discrepancies and comprehend the respondents' views. Tables 3, 4 illustrate the total scores for each of the eight competencies by age group and years of experience, separated by those with and without training. The numerical values assigned to different categories of importance span from “Not at all Important = 1” to “Very Important = 4” for various competencies, denoting the perceived significance of each competency. A higher numerical value implied a greater importance. If a competency consistently registers high scores across respondents, it could be a focal point for curriculum development or training programs.

**Table 3:** The total scores for each competency by age group and years of experience of PFA-trained disaster responders.

**Disaster responders with PFA training**						
**Age (years)**	**Year exp**	**Categories**	**4**	**3**	**2**	**1**
			**Very important**	**Important**	**Somewhat important**	**Not at all important**
16–30	1.0–13	Critical thinking	6	3	1	1
		Integrated problem-solving	7	2	2	0
		Systems thinking	6	4	1	0
		Anticipatory	5	5	1	0
		Normative	7	3	1	0
		Collaboration	6	4	1	0
		Strategic	7	1	3	0
		Self-awareness	7	2	1	1
31–40	3.0–10	Critical thinking	10	2	1	1
		Integrated problem-solving	10	3	1	0
		Systems thinking	11	2	0	1
		Anticipatory	10	4	0	0
		Normative	8	5	1	0
		Collaboration	9	4	0	1
		Strategic	8	5	1	0
		Self-awareness	10	3	1	0
41–50	1.0–16	Critical thinking	4	4	2	7
		Integrated problem-solving	4	4	7	2
		Systems thinking	4	4	6	3
		Anticipatory	4	4	6	3
		Normative	4	5	4	4
		Collaboration	5	5	5	2
		Strategic	5	4	6	2
		Self-awareness	5	3	7	2
51–60	2.0–33	Critical thinking	3	4	0	0
		Integrated problem-solving	3	4	0	0
		Systems thinking	2	5	0	0
		Anticipatory	2	5	0	0
		Normative	1	6	0	0
		Collaboration	2	5	0	0
		Strategic	1	6	0	0
		Self-awareness	3	4	0	0

**Table 4:** The total scores for each competency by age group and years of experience of disaster responders without PFA training.

**Disaster responders without PFA training**						
**Age (years)**	**Year exp**	**Categories**	**4**	**3**	**2**	**1**
			**Very important**	**Important**	**Somewhat important**	**Not at all important**
16–30	0.5–12	Critical thinking	5	1	0	0
		Integrated problem-solving	6	0	0	0
		Systems thinking	6	0	0	0
		Anticipatory	5	1	0	0
		Normative	5	1	0	0
		Collaboration	5	1	0	0
		Strategic	6	0	0	0
		Self-awareness	6	0	0	0
31–40	1–4	Critical thinking	2	0	0	0
		Integrated problem-solving	2	0	0	0
		Systems thinking	2	0	0	0
		Anticipatory	2	0	0	0
		Normative	2	0	0	0
		Collaboration	2	0	0	0
		Strategic	2	0	0	0
		Self-awareness	2	0	0	0
41–50	1–5	Critical thinking	2	1	0	0
		Integrated problem-solving	2	1	0	0
		Systems thinking	2	1	0	0
		Anticipatory	2	1	0	0
		Normative	2	1	0	0
		Collaboration	2	1	0	0
		Strategic	2	1	0	0
		Self-awareness	2	1	0	0
51–60	0.5–4	Critical thinking	1	3	0	0
		Integrated problem-solving	1	3	0	0
		Systems thinking	1	3	0	0
		Anticipatory	1	3	0	0
		Normative	2	2	0	0
		Collaboration	2	2	0	0
		Strategic	1	3	0	0
		Self-awareness	2	2	0	0

Furthermore, the data enables a comparative perspective of different competencies, underscoring the ones perceived as most and least important. It presents a structured methodology to grasp the collective perception of the importance of various competencies that can be pivotal in directing decision-making processes in relevant domains. The computation of statistical metrics such as mean, standard deviation, and variance, contributing to a more intricate understanding of the data set, was facilitated. Table 5 describes a descriptive analysis of the eight sustainability competencies, as evidenced by mean scores consistently above the 3.0 threshold. The mean values indicate the central tendency for each competence, suggesting a robust indication of the evaluated competencies. A lower value for standard deviation (Std) indicates a higher uniformity in the assessment. Correlation coefficients indicate a strong positive linear relationship with an undetermined variable of interest. At the same time, the variance quantifies the dispersion of the data around the mean, with more miniature figures indicating a tighter cluster of responses. The correlation analysis between each competency is demonstrated in Figures 4, 5, which examines the interrelationships between a set of sustainability competencies.

**Table 5:** The descriptive analysis of the sustainable competencies.

	**Std. dev**	**Variance**	**Mean**	**Correlation analysis**
**Descriptive analysis of disaster responders with PFA training**				
Critical thinking	1.14546	1.31207	3.02041	1.00000
Integrated problem-solving	0.91241	0.83248	3.20408	0.95276
Systems thinking	0.96495	0.93112	3.16327	0.88280
Anticipatory	0.89784	0.80612	3.16327	0.82724
Normative	0.92720	0.85969	3.12245	0.82146
Collaboration	0.88928	0.79082	3.20408	0.81392
Strategic	0.88976	0.79167	3.14286	0.83517
Self-awareness	0.95698	0.91582	3.20408	0.87037
**Descriptive analysis of disaster responders with no PFA training**				
Critical thinking	0.48795	0.23810	3.66667	1.00000
Integrated problem-solving	0.45774	0.20952	3.73333	0.85280
Systems thinking	0.45774	0.20952	3.73333	0.85280
Anticipatory	0.48795	0.23810	3.66667	1.00000
Normative	0.48795	0.23810	3.66667	0.70000
Collaboration	0.45774	0.20952	3.73333	0.85280
Strategic	0.45774	0.20952	3.73333	0.85280
Self-awareness	0.41404	0.17143	3.80000	0.70711

### Ethical consideration

This paper adheres strictly to the ethical guidelines and standards of academic research. All data and resources utilized in this study have been collected and used responsibly and ethically. The research methodology was designed to ensure the utmost respect for the privacy, confidentiality, and rights of any participants or sources involved. The necessary approval obtained from the Ritsumeikan Asia Pacific Univeristy compliance and ethics review committee and Fiji human health research and ethics review committee involving human subjects as outlined in the ethics statement.

## Results

The distribution of responses as per Table 3 with PFA training suggests that younger individuals (16–30 years) with less experience (0.5–13 years) tend to rate most of the competencies as “very important” and “important,” perceiving that they are essential for their profession as disaster responders. In contrast, the age group of 31–40 years with 3–10 years of experience showed a similar pattern of high respect for these competencies but with a slight increase in the “very important” category, with systems thinking, critical thinking, integrated problem solving, anticipatory and self-awareness receiving diverse responses which may reflect a significant understanding of these competencies as they gain experience. The data for the age group 41–50 years with considerable experience (1.0–16 years) represent a considerable shift to “somewhat important” and “not at all important.” This observation may imply that although these competencies are essential, their criticality varies depending on individual career stages and professional roles, suggesting a nuanced, role-specific interpretation of these skills. This finding indicates that professionals in the oldest age group (51–60 years), with substantial experience ranging from 2 to 33 years, exhibit a significant agreement in their assessment, primarily rating all the competencies as “very important” and “important.” This consensus among disaster responders may reflect a matured perspective on the vital role these competencies play in long-term career development and success.

The distribution of responses as per Table 4 without PFA training suggests that younger individuals (16–30 years) with less experience (0.5–12 years) tend to rate all of the competencies as “very important” and “important,” perceiving that they are essential for their profession as disaster responders. The majority of the respondents consider integrated problem-solving, strategic thinking, self-awareness, and systems thinking to be very important. The data presents a compelling narrative regarding the valuation of competencies across different age groups with varying experience levels. In the 31–40 age group, individuals with 1–4 years of experience uniformly rated all competencies—critical thinking, integrated problem-solving, systems thinking, anticipatory, normative, collaboration, strategic, and self-awareness—as “very important.” This unanimous rating suggests an insightful recognition of the importance of these competencies in the early stages of their career development.

On the other hand, the age group of 41–50 years, with a slightly broader experience range of 1–5 years, shows evidence of a shift in their appraisal. While still recognizing the importance of these competencies, there was a noticeable increase in the rating of “important” alongside “very important” across all competencies. This variation could indicate an evolving understanding of these competencies, where disaster responders with slightly more experience begin to differentiate the relative importance of these competencies based on their practical work experiences and type of disaster responses. The data concerning the oldest age group, 51–60 years, with experience ranging from 0.5 to 4 years, reveals a distinct trend in assessing competencies. Unlike the younger group, this cohort predominantly rated most competencies, such as critical thinking, integrated problem-solving, systems thinking, anticipation, and strategic thinking, as “important,” with a lesser emphasis on rating them as “very important.” Notably, there is an equal split between “very important” and “important” ratings for normative, collaboration, and self-awareness competencies. This pattern may suggest a more rational perspective on these competencies, possibly reflecting a refined understanding shaped by disaster experiences. It indicates a recognition of the importance of these competencies, yet with a judgment that balances principle with the experiences encountered over a longer career span.

**Figure 2:**
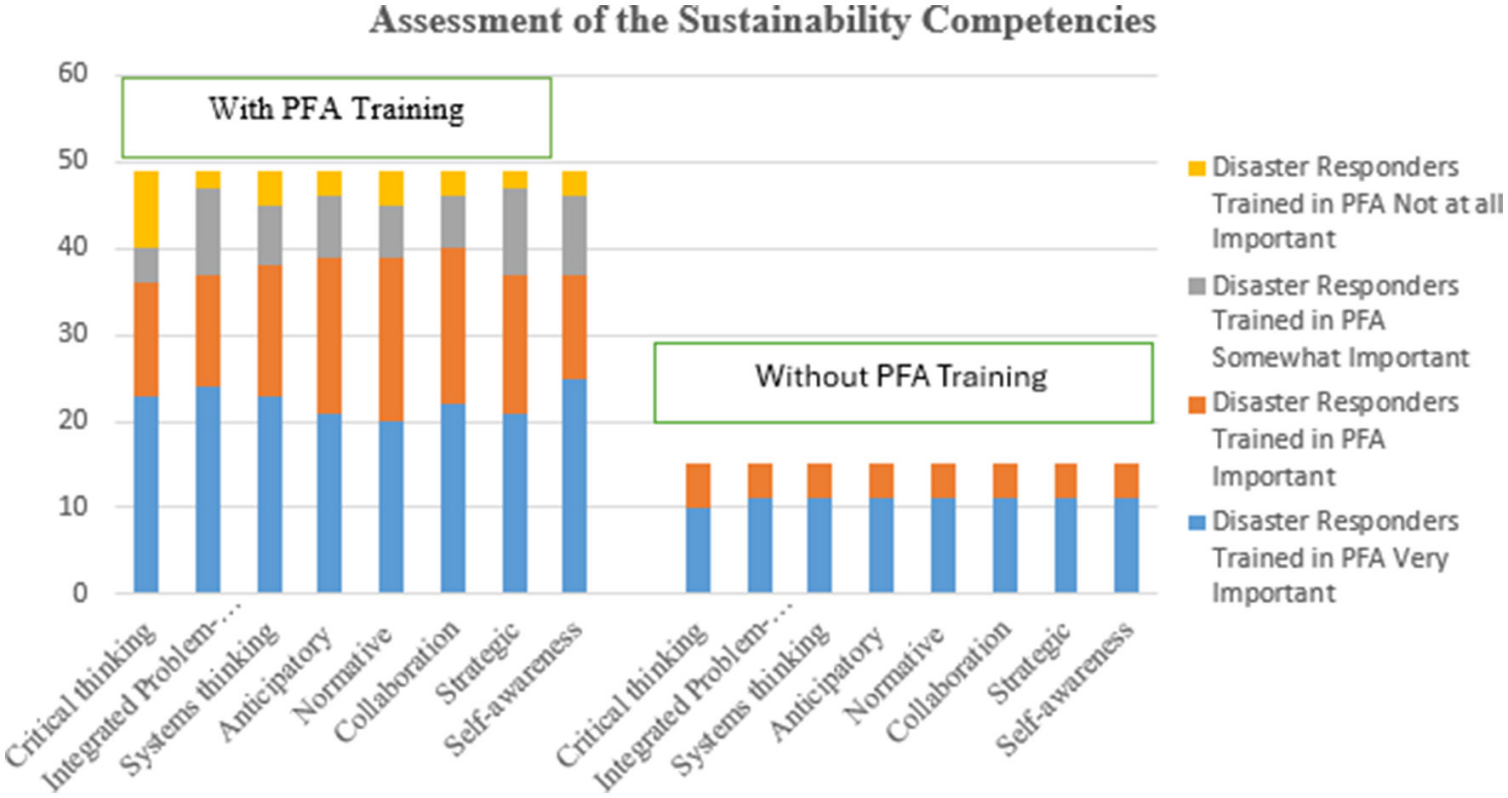
The assessment of the sustainability competencies.

Figure 2 represents the perceived importance of diverse sustainability competencies engaged in disaster response with and without PFA training. The data reveals that respondents with PFA training place a definite value on integrated problem-solving and self-awareness as the most frequently rated as “very important,” with self-awareness being the outstanding skill with 51% of the PFA trained participants. Critical thinking and systems thinking also command substantial recognition, with a majority deeming them “very important” or “important,” highlighting the exceptional place on cognitive capabilities and strategic insight within sustainability efforts.

Anticipatory and Strategic competencies, while deemed crucial, display a more balanced distribution of importance across respondents, with about 43% classifying them as “very important,” indicating a varied consensus on their integral role in sustainability. Normative competencies, conversely, demonstrate a slightly diminished attribution of “very important,” signaling a divergent perception of their significance in sustainable practice. However, it is noteworthy that a segment of respondents deems specific competencies as “not at all important,” with critical thinking sporadically perceived as such, although rarely.

For those without PFA training, the participants highly demonstrated all competencies as “very important” with substantial response to “important.” None demonstrated “somewhat important” or “not at all important.”

In contrast, those without PFA training uniformly acknowledge all competencies as “very important,” with a robust inclination toward “important,” and notably. This univocal recognition underscores the comprehensive competencies deemed essential for effectively navigating the intricate challenges of PFA intervention.

The descriptive analysis of disaster responders with PFA training demonstrates that integrated problem-solving, collaboration, and self-awareness competencies have the highest mean (Table 5), indicating that disaster responders consider these competencies more positive and most important. The overall mean for each competency is higher than 2, thus indicating that all eight competencies are accepted and that assessments are underpinned by elevated correlationand tight variances, suggesting a uniform evaluation and a distinct relational strength to the primary variable under consideration.

Equally, critical thinking for PFA-trained and anticipatory and normative for non-PFA trained exhibit the most significant variance despite a low mean, indicating a broader variance in participant responses. The data suggests that the responders possess substantial strengths across the evaluated competencies, with notable consistency in most domains. The slight deviation observed in critical thinking for those trained in PFA and self-awareness for those without PFA training may indicate a more subjective interpretation of this skill among the respondents.

**Figure 3:**
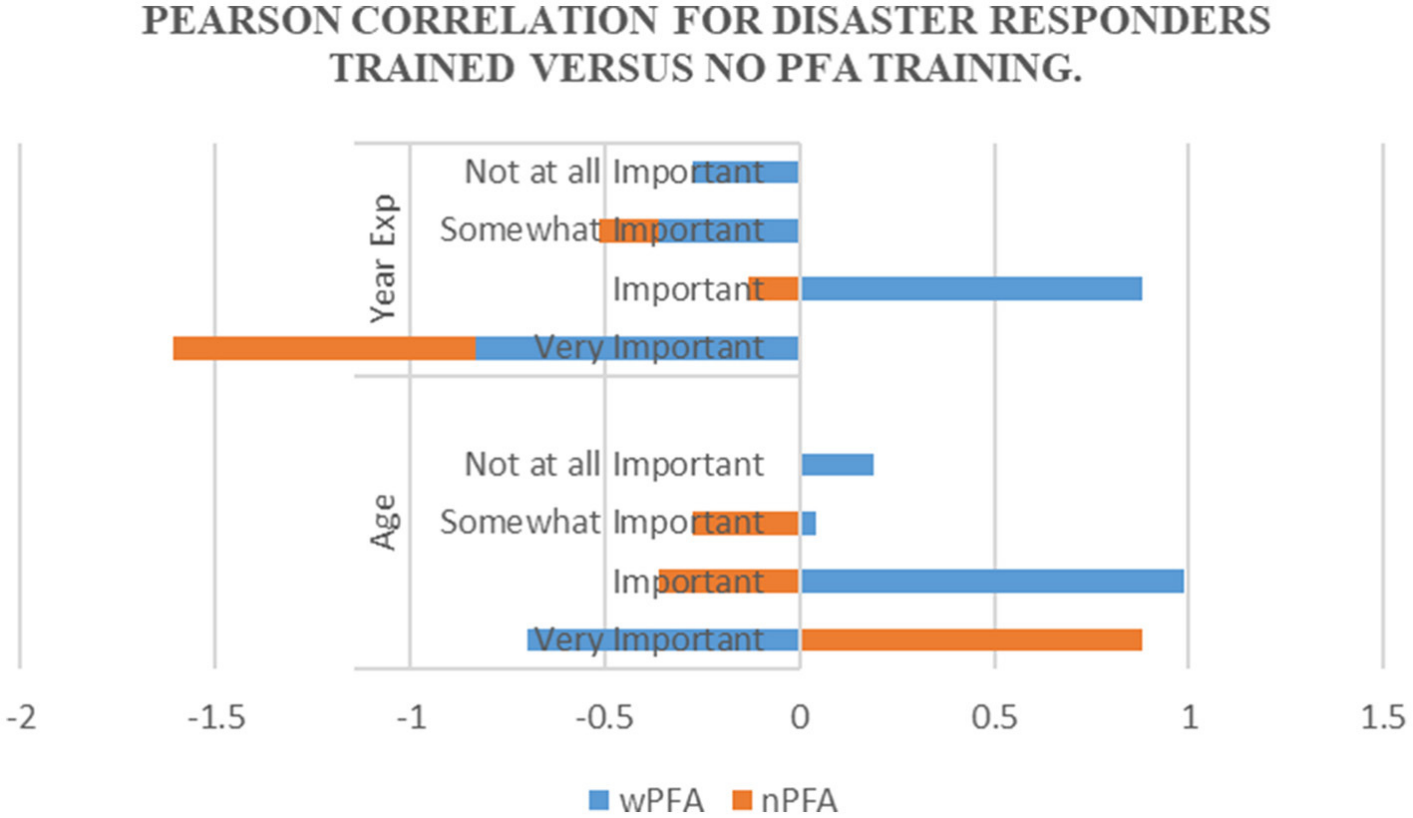
The correlation between age and years of experience.

While the propensity to rate elements as “very important” diminished slightly with age, there was a marginal increase (Figure 3) in classifying them as “important” or “not at all important.” A negative correlation with “very important” (−0.699) with PFA-trained participants and “important” (−0.359) and “somewhat important” (−0.276) with non-PFA-trained implies that the likelihood of rating something decreases somewhat as age increases. The negligible correlation with “somewhat important” for PFA-trained and non-PFA-trained ratings suggests an absence of a clear age-related trend. A positive correlation with “important” with PFA trained (0.989) indicates the likelihood of rating something as “important” increases with age. Meanwhile, the non-PFA trained showed a negative correlation (−0.359) on “important.” The data shows a weak correlation with “somewhat important” for PFA-trained (0.042) and non-PFA-trained (−0.276), suggesting an almost negligible linear relationship with age. A slightly more substantial but still weak positive correlation with “not at all important” for PFA trained (0.1916), hinting that there is a minor increase in the likelihood of rating something as “not at all important” with advancing age.

The correlation data interprets the perception of the impact of experience on disaster responders' valuation of competencies. The analysis reveals a negative correlation between years of experience and the likelihood of rating competencies as “very important” (−0.830) and “somewhat important” (−0.359) with those PFA-trained, suggesting that individuals with more experience may assign less criticality to these terms. Conversely, there is a strong positive correlation with the “important” for PFA-trained (0.883), indicating that as experience increases, so does the propensity to recognize competencies as necessary, even though not “very important.” The correlation with “not at all important” is negative (−0.276) for PFA-trained, though with a less pronounced significance, suggesting a modest increase in the likelihood that more experienced responders will regard competencies with this minor critical. The varying correlation values imply an apparent shift in perspective as respondents accumulate experience, with an inclination to avoid intense valuations in favor of more moderate assessments of competency importance.

In contrast, those with no PFA trained demonstrated a negative correlation for “very important” (−0,778), “important” (−0.133), and “somewhat important” (−0.156), respectively. Despite these trends, the correlations are not sufficiently robust to establish that experience is the ultimate interpreter of the importance ascribed to competencies. This complexity in correlation suggests that while experience informs perceptions, it does not influence how disaster responders evaluate the importance of competencies, indicating that other factors may also play a significant role in shaping these assessments within the field of disaster response.

**Figure 4:**
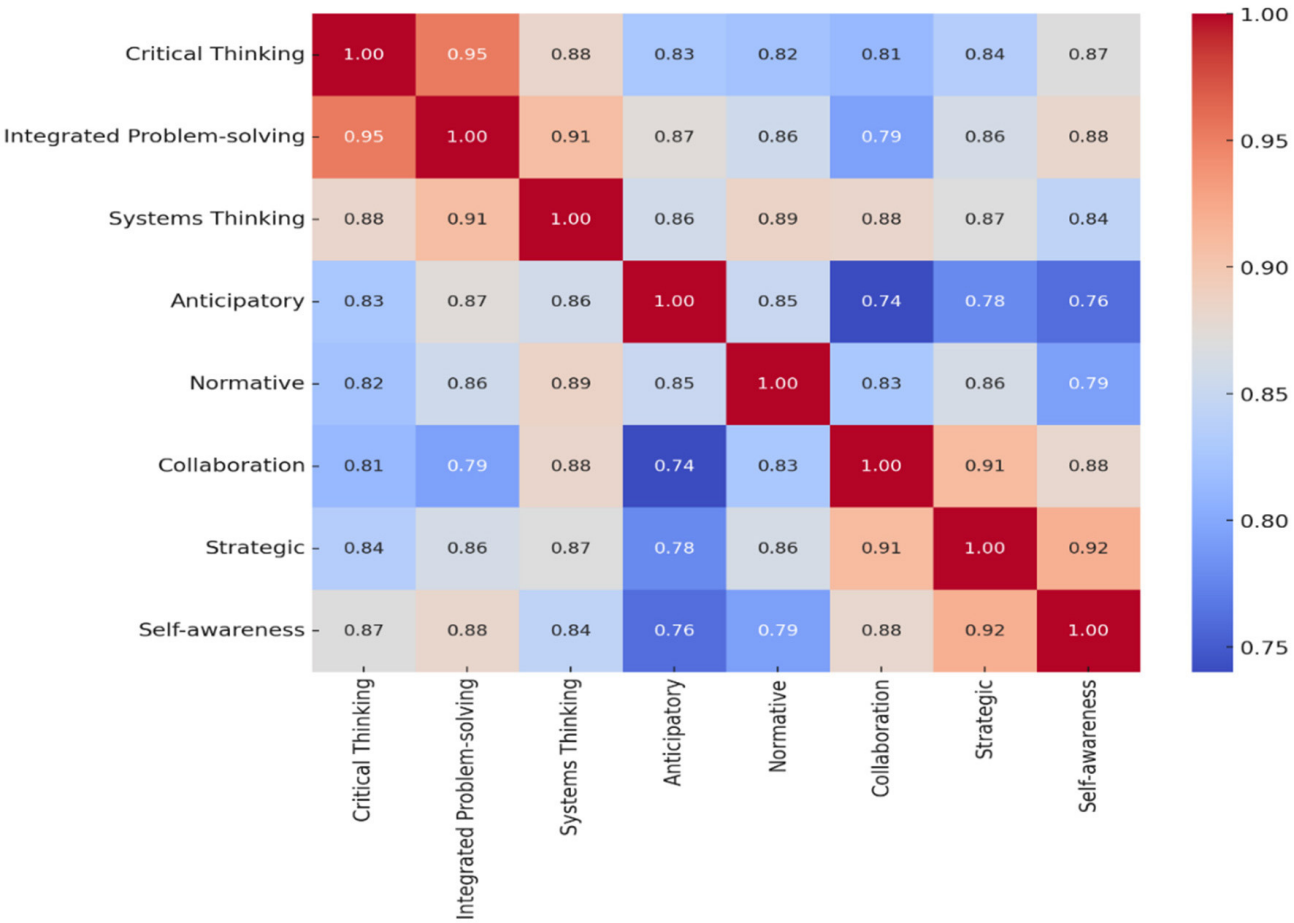
The correlation matrix between each sustainability competency and PFA-trained participants.

**Figure 5:**
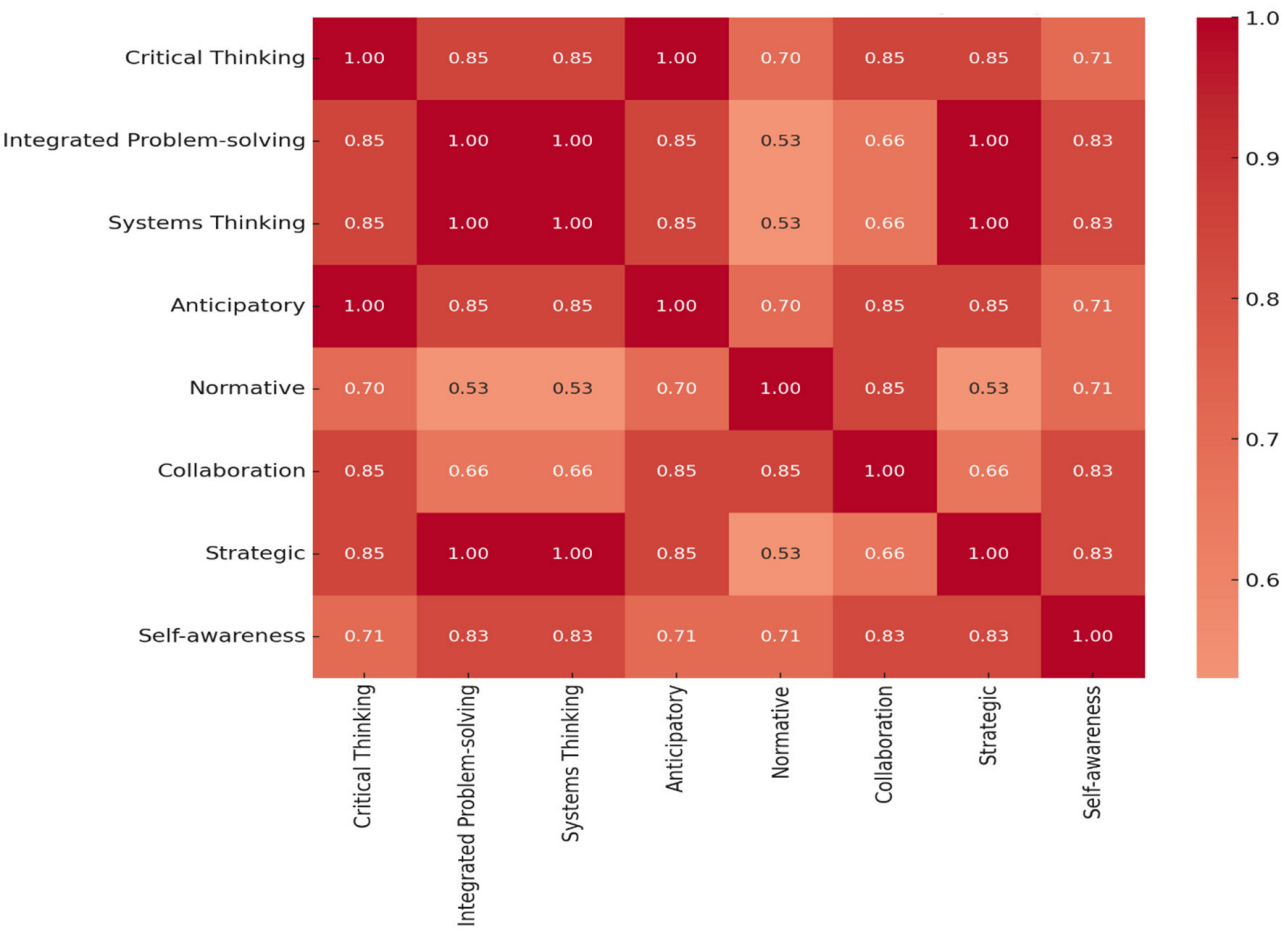
The correlation matrix between each sustainability competency (without PFA training).

The correlation matrix in Figures 4, 5 offers an insightful examination of the interrelationships between a set of sustainability competencies. The matrices quantify the linear relationships between competency pairs, with the correlation coefficients ranging from −1 to 1. A coefficient approaching 1.00 denotes a robust positive relationship, as seen between integrated problem-solving and critical thinking, which exhibits a strong correlation of 0.95 in Figure 4, suggesting substantial cognitive overlap. Moreover, strategic planning and self-awareness display a notable correlation of 0.92, indicating these competencies share synergistic cognitive functions vital for tackling complex issues. This suggests a significant overlap in the cognitive processes that these competencies engage.

The pattern of interdependency underscores the critical role of systemic interrelationships in collaborative sustainability efforts. For instance, the close correlation of 0.91 between systems thinking and integrated problem solving points to a collaborative relationship essential for complex cognitive and interpersonal operations in scenarios such as PFA interventions.

The corrected correlation matrix per Figure 5 reflects the relationships between sustainability competencies for those without PFA training. The matrix shows varying degrees of correlation, ranging from moderate to high, indicating how these competencies are perceived to interrelate. High values (closer to 1) suggest a strong positive relationship, indicating that as one competency is deemed significant, others are likely viewed similarly.

Conversely, Figure 5 illustrates the dynamics for those without PFA training, where the spectrum of correlations is consistently positive. This positive association implies that the enhancement of one competency is likely to co-evolve with others. However, the matrices also reveal weaker links, indicating stable interconnectivity across competencies. A correlation of 0.53 between normative and integrated problem-solving, systems thinking, and strategic planning reflects a potential divergence in the perceived relevance or application of these competencies among individuals lacking PFA training.

Analyzing both correlations of sustainability competencies reveals a landscape of interrelated skills necessary for effective sustainability practice. The first matrix indicates a strong positive correlation among competencies such as integrated problem-solving, systems thinking, critical thinking, collaboration, and self-awareness, suggesting these areas are often simultaneously valued. The interpretation could imply that individuals who excel in one of these competencies will likely be proficient in others, pointing to a cluster of cognitive skills that reinforce each other in sustainability-related decision-making processes (19).

In contrast, the second matrix highlights varied degrees of correlation, with specific competencies like normative demonstrating a less consistent but still significant relationship with others, thus the perception of normative as a standalone skill that complements but is not entirely dependent on other competencies.

Collectively, the results from both matrices suggest that while core competencies are intrinsically linked, reflecting a consistent skill set, distinct competencies contribute uniquely to the sustainability skill portfolio. A comprehensive approach to sustainability education and PFA training fosters the development of interrelated cognitive skills and enhances individual competencies that can independently drive sustainable outcomes (20).

## Discussion

### The significance of sustainability competencies in evaluating the effectiveness of psychological first aid

This study underscores the intricate correlation between PFA principles and sustainability competencies, revealing critical insights for developing and refining the PFA curriculum. While PFA traditionally emphasizes immediate psychological responses (12), and sustainability focuses on long-term outcomes (19), their convergence is noteworthy, especially in fostering psychological resilience and adaptive capacities for sustainable development. The roles that involve disaster response, especially regarding climate change, require critical thinking to understand the complexity of environmental and context-based challenges. The analysis of sustainability competencies among disaster responders reveals a complex interplay between experience, age, and PFA training. Younger responders and those with less experience tend to rate all competencies as “very important” or “important,” indicating a perception of these skills as essential to their roles. As experience increases, there is a discernible shift in how competencies are valued, with a more nuanced understanding emerging among those in the 31–40 and 41–50 age groups. Integrating sustainability competencies into the PFA theoretical framework is pivotal for shaping effective interventions. For relevance and effectiveness, creating a connection between sustainability skills and the responsibilities of disaster response staff and volunteers is essential, ensuring that these competencies align with the intended outcomes of sustainable PFA practices (29). Responders with PFA training particularly value integrated problem-solving, collaboration, and self-awareness, while critical thinking and systems thinking are also recognized but with more variance in responses. Correlation matrices show that specific competencies are strongly interrelated, suggesting that proficiency in one area may predict proficiency in others. However, the correlation is less pronounced among those without PFA training, indicating that while experience informs perceptions, it is not the sole determinant of competency valuation. The data underscores the importance of a comprehensive approach to sustainability education and PFA training to enhance the cognitive skills necessary for effective disaster response. These competencies also encompass understanding climatic and disaster risks, emergency response, disaster management, effective communication, and adaptability, acknowledging the complex societal, cultural, and environmental factors influencing mental health (30). This integration addresses a significant demand for sustainable competencies in PFA interventions, aiming to bridge the gap between current curricula and the evolving needs of disaster responders.

The results reveal significant insights into various sustainability competencies. The respondents, predominantly female demonstrated strong skills across eight competencies, with mean scores above 3.0, indicating robust evidence of the competencies. The data, for instance, suggest that female responders actively engage in roles that anticipate the psychosocial impacts of disasters and plan for long-term recovery. Integrating sustainability competencies with PFA principles can provide a holistic approach to disaster response, enhancing the effectiveness of interventions and supporting the resilience of communities and responders alike. For example, critical thinking improves the assessment of psychological needs, while anticipatory skills ensure preparedness for mental health crises. Disaster management necessitates reasoning to ascertain predicaments, comprehend presumptions, assess contentions, and employ logical thought. These proficiencies are indispensable for the administration of disaster response and rehabilitation, as well as the augmentation of a leader's assurance, credibility, and influence during crises that affect an entire community (31). Collaborative competencies facilitate resource sharing, and integrated problem-solving allows for comprehensive strategies to address the psychosocial aspects of disasters. The anticipatory abilities are equally crucial for developing strategies for future disasters. The results show a potential trend in which the assessment of competencies shifts from being considered very important in early career phases to being more diverse as individuals gain experience and presumably advance in their careers as disaster responders. Male responders' involvement across logistics, human resources, and finance during disaster response underscores the need for collaborative efforts in disaster management. Emphasizing emergency response techniques within the curriculum is vital. Educators in Fiji highlighted the importance of contextual and field-based learning in leadership and management training, suggesting its potential applicability in PFA training (32).

Self-awareness ensures that disaster responders maintain their wellbeing and provide sustained, empathetic support to disaster-affected populations (24). Our results suggest that male and female responders are involved in areas that could benefit from improved self-awareness to prevent compassion exhaustion. Self-awareness significantly encourages the competencies of responders by fostering an understanding of their emotional, cognitive, and behavioral responses in high-pressure scenarios (4, 33). Improved self-awareness enables responders to effectively regulate stress and emotions and maintain composure and concentration during crises. Acknowledging personal biases promotes a more open and impartial approach to emergencies. Awareness of individual strengths and weaknesses enables disaster responders to seek additional support or resources and optimize the effectiveness of their assistance (4). Self-awareness enhances communication skills, allowing responders to moderate emotional expressions and interact with clarity and empathy (33).

Integrated problem-solving skills emerged as a critical skill, especially for roles that intersect various areas of disaster response. Respondents who engage in community-based health, first aid, and disaster resilience activities illustrate the need for problem-solving skills that synthesize knowledge from different regions to address complex real-world problems. Respondents play critical roles in supporting individuals by evaluating needs and administering interventions (4). Their tasks include implementing basic PFA principles: ensuring safety, providing comfort, and providing practical help (22). They are instrumental in connecting individuals with supplementary support services, including mental health professionals and community resources, with the support they need (33). Integrating problem-solving approaches and diverse assessment methods, such as practical scenarios and reflective exercises, is essential for quantifying learning outcomes and ensuring competency-based learning (4, 34). A small-scale study in Fiji demonstrates the benefits of reflective exercises in enhancing learners' capacity to generate new knowledge and apply theoretical constructs in practical scenarios (32). Learners engaged in role-playing exercises strengthens collaboration and communication skills, alternating roles as PFA recipients, providers, and observers (24). Collaboration ensures a coherent response across different facets of a disaster, facilitating a unified approach that leverages the strengths of various sectors. With increased experience, disaster responders may find some competencies less critical than they initially thought, or it could reflect a reorientation of the importance of skills when they transition through different roles or industries and have acquired these competencies. Disaster management collaboration is essential to improve response effectiveness and efficiency, involving various organizations and stakeholders. It facilitates the exchange of information, resource pooling, and improved decision-making, which is crucial for dealing with complex disaster scenarios (35). Collaboration leads to more comprehensive management strategies like integrated problem-solving competence and community engagement. Furthermore, it aids in capacity building, knowledge transfer, and developing compelling education and training programs, ultimately improving resilience and outcomes for affected communities. The results could likewise suggest that younger disaster responders view these competencies as essential to gaining a competitive edge and establishing themselves in their roles.

On the other hand, older and more experienced disaster responders could rely more on specialized knowledge or other skills not included in the survey, such as leadership or domain-specific skills. It is crucial to consider the role of external factors such as the availability of resources in a particular organization, the level of demands in the diverse culture, and socio-economic factors that could significantly influence the perception of the importance of these competencies over time. Additionally, the data could reflect generational attitudes toward work and professional development. Implementing critical reflection, as evidenced by a curriculum development course in Fiji, reveals challenges due to cultural and colonial influences (32). However, fostering an inclusive and safe classroom environment that encourages reflective discussions and peer feedback can significantly enhance critical thinking and mutual learning among trainees (34). This approach is instrumental in cultivating a deeper understanding of PFA principles and their application. This context-specific approach is particularly pertinent in Pacific Island societies, where social hierarchies may impede knowledge transfer.

Tailoring learning outcomes to specific objectives within the PFA curriculum is crucial. The study's findings emphasize competencies such as integrated problem-solving, systems thinking, critical thinking, anticipatory skills, normative understanding, strategic thinking, self-awareness, and collaboration (19) in curriculum development. These competencies enable PFA providers to deliver more nuanced and culturally appropriate support, optimizing resource allocation and intervention strategies (30). An academic study emphasizes integrating Education for Sustainable Development (ESD) principles across all educational levels, as the 2030 Agenda for Sustainable Development outlines. It advocates for recognizing ESD as a core component of quality education in diverse settings, from preschools to higher education and informal learning environments. It stresses the importance of creating and validating assessment tools to link sustainability competencies with employability and educational quality assurance (29). Our research underscores the importance of sustainability competencies in enhancing PFA principles among disaster responders in Fiji inclusive of informal setting. The study reveals that the perceived significance of these competencies may vary with age and experience, reflecting a dynamic interaction of contextual factors rather than a decline in competence value. Integrating these competencies into disaster management training is vital for creating a comprehensive and adaptive response system, particularly in the face of escalating climatic events. A flexible curriculum accommodating diverse learning needs fosters engagement and addresses differing perspectives on sustainability competencies. This study emphasizes the need for holistic reform in the PFA curriculum, which addresses aligning and incorporating sustainability competencies as learning outcomes, thereby equipping responders with the diverse skills necessary for effective and sustainable disaster management.

Further research could explore these trends more deeply, considering the qualitative aspects of why specific competencies are rated differently across careers or years of experience. The study lays a foundation for further research into the reasons behind each competency category's perceived importance in PFA curricula. Additionally, the development of validated measurement tools tailored for assessing sustainable psychosocial support competencies adaptable to various cultural contexts remains a challenge. Such tools are crucial for evaluating the effectiveness of PFA practices in diverse settings.

## Conclusion

Integrating sustainability competencies into PFA curricula to enhance community and responder resilience is imperative. It advocates for a holistic, culturally responsive curriculum reform, ensuring PFA training is comprehensive and attuned to diverse needs. This study critically examined the correlation between sustainability competencies and PFA within Fiji's unique sociocultural context to enhance PFA training and intervention. Employing a theoretical framework and leveraging a cross-sectional survey, the study analyzed Fiji disaster responders' assessments to perceive the importance of sustainability competencies among Fiji disaster responders. Intercompetency correlations emerged, underscoring the symbiosis of these competencies in effective PFA deployment.

The study highlights a multifaceted correlation between age, experience, and the valuation of professional competencies among disaster responders. The appraisal patterns across different age groups, especially among those with PFA training, suggest a tendency to moderate assessments of competency importance with increasing experience. This interplay between age, experience, and competency valuation underscores the diverse factors influencing perceptions in the disaster response field beyond experience alone. The results show that sustainability competencies can be the ultimate to the effectiveness of PFA measurement and interventions. However, the research is lessened by its localized scope and the absence of primary empirical data, which may limit the applicability of its conclusions. The limitations include its context-specific focus on Fiji, which may affect the generalizability of findings to different geographical and cultural contexts. In addition, the lack of primary empirical data, such as direct feedback or interviews with Fiji's disaster responders, limits the study's ability to provide insights into the practical application of these competencies in PFA in a different context.

The outcome lays the groundwork for developing culturally relevant competency assessment tools, essential for refining PFA strategies to effectively address the varied needs of disaster-impacted populations. It underlines the necessity of a distinctive, adaptable PFA framework, incorporating sustainability competencies as fundamental components, and emphasizes the importance of validated measurement tools for assessing these competencies in mental health and psychosocial support across different cultural contexts. This approach is crucial for both competency-based evaluations of the effectiveness of PFA practices and customizing them to meet the diverse requirements of those affected by disasters.

## Data availability statement

The original contributions presented in the study are included in the article/Supplementary material, further inquiries can be directed to the corresponding author.

## Ethics statement

The studies involving humans were approved by the necessary approval (approval number 2022-7) and was obtained from the research compliance and Ethics Review Committee of the Ritsumeikan Asia Pacific University on research involving human subjects and the Fiji Human Health Research and Ethics Review Committee (FNHRERC number 11/2023). The studies were conducted in accordance with the local legislation and institutional requirements. The ethics committee/institutional review board waived the requirement of written informed consent for participation from the participants or the participants' legal guardians/next of kin because the survey questionnaire had a clause for informed consent; if the participants did not wish to participate, they had the right not to answer the survey. Those who participated gave consent by answering the survey.

## Author contributions

MN: Conceptualization, Data curation, Formal analysis, Funding acquisition, Investigation, Methodology, Project administration, Resources, Validation, Visualization, Writing—original draft, Writing—review & editing. SM: Funding acquisition, Resources, Supervision, Validation, Visualization, Writing—review & editing.
